# Measurement properties of patient-reported outcome measures (PROMs) in hyperhidrosis: a systematic review

**DOI:** 10.1007/s11136-021-02958-3

**Published:** 2021-07-30

**Authors:** Michaela Gabes, Helge Knüttel, Gesina Kann, Christina Tischer, Christian J. Apfelbacher

**Affiliations:** 1 Institute of Social Medicine and Health Systems Research, Otto-Von-Guericke-University Magdeburg, Magdeburg, Germany; 2 Medical Sociology, Department of Epidemiology and Preventive Medicine, University of Regensburg, Dr.-Gessler-Str. 17, 93051 Regensburg, Germany; 3 University Library, University of Regensburg, Regensburg, Germany; 4 State Institute of Health, Bavarian Health and Food Safety Authority, Bad Kissingen, Germany

**Keywords:** Hyperhidrosis, Patient-reported outcome measures, Measurement properties, Validity, Reliability, Responsiveness

## Abstract

**Purpose:**

To critically appraise, compare and summarize the quality of all existing PROMs that have been validated in hyperhidrosis to at least some extend by applying the COnsensus-based Standards for the selection of health Measurement INstruments (COSMIN) methodology. Thereby, we aim to give a recommendation for the use of PROMs in future clinical trials in hyperhidrosis.

**Methods:**

We considered studies evaluating, describing or comparing measurement properties of PROMs as eligible. A systematic literature search in three big databases (MEDLINE, EMBASE and Web of Science) was performed. We assessed the methodological quality of each included study using the COSMIN Risk of Bias checklist. Furthermore, we applied predefined quality criteria for good measurement properties and finally, graded the quality of the evidence.

**Results:**

Twenty-four articles reporting on 13 patient-reported outcome measures were included. Three instruments can be further recommended for use. They showed evidence for sufficient content validity and moderate- to high-quality evidence for sufficient internal consistency. The methodological assessment showed existing evidence gaps for eight other PROMs, which therefore require further validation studies to make an adequate decision on their recommendation. The Hyperhidrosis Disease Severity Measure-Axillary (HDSM-Ax) and the short-form health survey with 36 items (SF-36) were the only questionnaires not recommended for use in patients with hyperhidrosis due to moderate- to high-quality evidence for insufficient measurement properties.

**Conclusion:**

Three PROMs, the Hyperhidrosis Quality of Life Index (HidroQoL), the Hyperhidrosis Questionnaire (HQ) and the Sweating Cognitions Inventory (SCI), can be recommended for use in future clinical trials in hyperhidrosis. Results obtained with these three instruments can be seen as trustworthy. Nevertheless, further validation of all three PROMs is desirable.

**Systematic review registration:**

PROSPERO CRD42020170247

## Background

Hyperhidrosis is characterized by excessive sweating beyond physiological needs. This disorder can be generalized, involving the whole body, or focal, involving specific areas of the body such as the axillae (axillary hyperhidrosis), the hands and feet (palmar and plantar hyperhidrosis) or the face (cranio-facial hyperhidrosis) [8, 49, 50]. With a recently found prevalence of 4.8% in the USA, about more than half of the affected individuals suffer from axillary hyperhidrosis [8, 42]. The severity of hyperhidrosis can range from light sweating to real dripping. Therefore, those affected often report negative impacts on their quality of life (QoL) including for example limited daily activities, less social relationships, impairments in their study or work life, and a general reduced emotional well-being [8, 14, 19].

Measurement instruments that try to capture what is reported by affected individuals are called “patient-reported outcome measures (PROMs)”. PROMs are self-completed questionnaires reflecting the patient’s perspective and measuring, e.g. severity or QoL. By PROMs, the involvement of patients in both clinical research as well as routine health care can be fostered [28, 51].

Several PROMs that cover diverse constructs have been developed and reported in the literature for patients with hyperhidrosis, for instance, the Hyperhidrosis Quality of Life Index (HidroQoL) [20], the Hyperhidrosis Disease Severity Scale (HDSS) [47] or the Axillary Sweating Daily Diary (ASDD) [34]. In clinical research and practice, not only hyperhidrosis-specific PROMs are used but also skin-specific or more generic PROMs such as the Dermatology Life Quality Index (DLQI) [9] or the short-form health survey (SF-36) [18, 38].

Especially in clinical research, it is important to select measurement instruments with sufficient measurement properties in the population of interest. PROMs should be reliable, valid, responsive and feasible. The selection of instruments should be based on complete information regarding these measurement properties and the quality of the underlying research.

In preparation of the development of the HidroQoL, Kamudoni rated the psychometric properties of several instruments used in measuring QoL in hyperhidrosis. For this rating, he used literature-based standard quality criteria [18]. Wade et al*.* [50] also conducted a review of the most commonly used QoL measurement instruments in hyperhidrosis, but explicitly refrained from using the COnsensus-based Standards for the selection of health Measurement INstruments (COSMIN), as this would have been beyond the scope of their review given the high level of detailed information required and the level of expertise needed in the application of the COSMIN checklist.

We intend to fill this gap by performing a systematic comparison of all existing PROMs in hyperhidrosis (not just of those measuring QoL) and an assessment of the quality of these PROMs using the established COSMIN methodology.

## Objectives

Our main objective was to critically appraise, compare and summarize the quality of all existing PROMs that have been validated in hyperhidrosis to at least some extent by applying COSMIN methodology.

More specifically, our objectives were

1. to systematically assess the measurement properties of PROMs in hyperhidrosis and
2. to identify PROMs in hyperhidrosis
  - that meet the predefined criteria to be recommended in future hyperhidrosis trials;
  - that have the potential to be recommended in the future depending on the results of further validation studies;
  - that do not meet the predefined criteria to be recommended and therefore should not be used anymore.

## Materials and methods

### Protocol and registration

The methods of this systematic review were developed in accordance with the Preferred Reporting Items for Systematic Reviews and Meta-Analyses Protocols (PRISMA-P) statement [40]. The corresponding study protocol was registered in the International Prospective Register of Systematic Reviews (PROSPERO): CRD42020170247 and published in *Systematic Reviews* [10].

### Literature search

A systematic, librarian assisted literature search was performed in the bibliographic databases MEDLINE (via Ovid, 1946–02 June 2020, database code “medall”), EMBASE (via Ovid, 1974–02 June 2020, database code “oemezd”), Science Citation Index Expanded (1965–02 June 2020, database code "SCI-EXPANDED") and Social Sciences Citation Index (1990–02 June 2020, database code "SSCI") (the latter two simultaneously via Web of Science) on 02 June 2020 with a last update on 11 June 2021. The search strategy comprised the following search elements [32]:

A. Target population: Hyperhidrosis. In order to reach maximal sensitivity a broad compilation of controlled vocabulary and free text terms was used. The search strategy for this element was not peer reviewed.
B. Construct of interest: All patient-reported outcome measures regardless of the underlying construct. For optimal sensitivity the search strategy of this search element was based on a combination of the PubMed filter "Quality of life (QoL)" of Vissers and de Vries [48], the PubMed filter "Patient reported outcome measures (PROMs)" of Jansma and de Vries [17], and additional search terms from the "PROM group construct & instrument type filter" of Mackintosh et al*.* [27] Patient-reported outcome measures is a broad term and it includes measures of QoL or health status [12, 28].
C. Measurement properties: The validated and sensitive search filter (recommended by the COSMIN group [36]) for finding studies on measurement properties developed by Terwee et al*.* [44] was used. We employed the translation of the original PubMed filter to Ovid MEDLINE by Alberta University [4].
D. Feasibility of PROMs: The search strategy for this element was based on the search terms for the concept ‘feasibility’ of Heinl et al*.* [15] (included in their search statement #1, additional file 2).
E. Individual PROMs: A list of known relevant PROMs including those identified in the preliminary work of Kamudoni [18] and in the systematic review of Wade et al. [50]
F. Exclusion filter: This was the exclusion filter from Terwee et al*.* [44] for a number of irrelevant publication types and for animal-only studies.

The search elements were combined as follows in order to identify all articles on the measurement properties or the feasibility of PROMs in hyperhidrosis. From these records, the exclusion filter removed irrelevant publication types as well as animal-only studies: ((A AND B AND (C OR D)) OR (C AND E)) NOT F, or in words: ((population AND construct AND (measurement properties OR feasibility)) OR (individual PROMs AND measurement properties)) NOT (exclusion filter).

Search strategies for MEDLINE, EMBASE and Web of Science were developed. The initially developed MEDLINE search strategy was translated to the other databases choosing appropriate syntax and index terms. The full, reproducible search strategies are included in Appendix 1 (supplementary files). A PRISMA-S checklist is in Appendix 2 (supplementary files) [37].

In addition, databases specific for PROMs were searched for records relevant to the target population: PROQOLID (https://eprovide.mapi-trust.org/about/about-proqolid), the COSMIN database of systematic reviews of outcome measurement instruments (http://www.cosmin.nl/database-of-systematic-reviews.html), the Test Archive of Leibniz Institute for Psychology Information (https://www.testarchiv.eu/) and the PubPsych search engine (https://pubpsych.zpid.de/pubpsych/). In addition to the electronic search, hand-searching was conducted by perusing reference lists of the studies included and by searching key articles on this topic. No study registries were searched due to the study designs eligible for this review. We did not contact persons or institutions in order to seek additional studies.

Subsequently, the bibliographic databases and the databases specifically on PROMs were searched again with the names of hyperhidrosis-specific PROMs found during the initial search.

There were no restrictions regarding publication date. Only papers in English, German, French or Italian were included. After the deduplication in EndNote X9 following the method of Bramer et al*.* [2], titles and abstracts were screened in EndNote. No further software was used for the full-text review. Data were extracted using excel sheets.

### Eligible studies

The eligibility criteria are in agreement with the COSMIN guideline for systematic reviews of patient-reported outcome measures [36]. The population of interest were patients with hyperhidrosis. At least 50% of the study sample need to consist of hyperhidrosis patients to fulfil the eligibility criteria. The evaluation of measurement properties, the development of a PROM or the evaluation of the interpretability of the PROMs of interest should be the principal aim of selected studies. Studies that only use the PROM to measure the outcome or in which the PROM is used for the validation of another instrument were excluded. Only full-text articles were included because abstracts or posters provide quite often very limited information on the design of a study. Studies that concern the development (“development paper”) and/or the evaluation of the measurement properties (“validation paper”) of PROMs were included as well (Table 1).

**Table 1:** Inclusion and exclusion criteria

	Inclusion criteria	Exclusion criteria
Population	Patients with hyperhidrosis	All other
Study design	PROM development study, validation study	All other study designs
Outcome	All patient-reported outcomes	Non-patient-reported outcomes, such as biomarkers or physiology of the skin
Type of measurement instrument	Patient-reported outcome measures	All others
Publication type	Articles with available full-text	Abstracts

*PROM* patient-reported outcome measure

### Study selection

Titles and abstracts found in the literature search were independently judged by two reviewers. For the remaining titles and abstracts, full-text articles were searched and judged for eligibility also by two reviewers independently. If any disagreement occurred, consensus was reached by consulting a third reviewer. If at least one reviewer considered a study as relevant based on the abstract, or in case of doubt, the full-text article was screened.

### Data extraction

#### Assessment of measurement properties and adequacy of the PROMs

Measurement properties were evaluated in the following order:

- Evaluation of the content validity.
- Evaluation of internal structure including structural validity, internal consistency and cross-cultural validity/measurement invariance.
- Evaluation of remaining measurement properties including reliability, measurement error, criterion validity, hypotheses testing for construct validity and responsiveness.

All measurement properties were evaluated following three sub steps, except for the measurement property “criterion validity” since no gold standard for PROMs in hyperhidrosis exists. For construct validity and responsiveness, we formulated hypotheses to evaluate the results against.

First, the methodological quality of the included studies was evaluated by two independent reviewers using the COnsensus-based Standards for the selection of health Measurement INstruments (COSMIN) Risk of Bias checklist which was developed exclusively for systematic reviews of PROMs [30]. Both reviewers had a psychological and therefore also psychometric background and were familiar with the COSMIN methodology. The COSMIN Risk of Bias checklist consists of 10 Boxes, each for one measurement property (Table 2). Only those boxes for the measurement properties that are assessed in an article were filled in.

**Table 2:** Boxes of the COSMIN Risk of Bias checklist [30]

Box 1	PROM development	Content validity
Box 2	Content validity	
Box 3	Structural validity	Internal structure
Box 4	Internal consistency	
Box 5	Cross-cultural validity\measurement invariance	
Box 6	Reliability	Remaining measurement properties
Box 7	Measurement error	
Box 8	Criterion validity	
Box 9	Hypotheses testing for construct validity	
Box 10	Responsiveness	

*COSMIN* COnsensus-based Standards for the selection of health Measurement Instruments, *PROM* patient-reported outcome measure

All measurement properties of the COSMIN Risk of Bias checklist are clearly defined [33]. Content validity is considered as the most important measurement property because the items of a PROM have to be relevant, comprehensive and comprehensible regarding the population and construct of interest [45]. If there is high-quality evidence for insufficient content validity, the PROM was not further assessed and directly categorized as C, i.e. the PROM should not be recommended for use. Each study was rated on a 4-point rating scale (that is, “inadequate”, “doubtful”, “adequate”, “very good”). The overall quality of a study was determined by the lowest rating of any standard in the box, i.e. “the worst score counts” principle [30]. Each study on a measurement property was assessed separately and all measurement properties of each study were rated as either very good, adequate, doubtful or inadequate [31].

Second, we extracted relevant data on characteristics of the included PROMs and the included study populations and summarized them in evidence tables [31]. Interpretability and feasibility which are also important for a recommendation were described after the evaluation of the measurement properties. Interpretability means the degree to which qualitative meaning can be assigned to a PROM’s quantitative score. Feasibility contains aspects of the ease of application (e.g. costs, length, ease of administration) [31].

Furthermore, we applied quality criteria. We used updated criteria for good measurement properties recommended by the COSMIN group [36]. The result of each single study was rated as either sufficient (+), insufficient (−) or indeterminate (?) [31].

Third, we aimed to summarize the evidence per measurement property per PROM, rate the overall result against criteria for good measurement properties and grade the quality of the evidence by the GRADE approach. Here, the focus was no longer on the single studies, but on the PROM [31].

The third substep included several further substeps: (1) we looked at the consistency of our results, searched for explanations if inconsistency occurred or downgraded for inconsistency if no explanation was found; (2) we pooled or summarized the results in Summary of Findings (SoF) Tables, each measurement property per PROM in one table; (3) we rated each pooled or summarized result again against the quality criteria to obtain an overall rating for the pooled or summarized result as either sufficient (+), insufficient (−), inconsistent ( ±) or indeterminate (?); and (4) we graded the quality of the evidence to define whether the pooled or summarized result was trustworthy [31]. The recognition of the quality of evidence can help to prevent misguided recommendations [13]. Using the GRADE approach, we determined whether confidence in estimates of true measurement properties is given. We used a GRADE approach with four GRADE factors (risk of bias, inconsistency, imprecision and indirectness) and four levels of quality evidence (high, moderate, low or very low) (Table 3). If the results did not seem trustworthy, the quality of evidence was downgraded. Each PROM was graded separately [36]. If the overall rating for a measurement property was inconsistent (±) or indeterminate (?), the quality of evidence was not graded [36].

**Table 3:** GRADE approach for grading the quality of evidence [36]

Quality of evidence	Lower if
High (We are very confident that the true measurement property lies close to that of the estimate of the measurement property)	Risk of bias: 1. Serious 2. Very serious 3. Extremely serious
Moderate (We are moderately confident that the true measurement property is likely to be close to the estimate of the measurement property, but there is a possibility that it is substantially different)	Inconsistency: 1. Serious 2. Very serious
Low (Our confidence in the measurement property estimate is limited: the true measurement property may be substantially different form the estimate of the measurement property)	Imprecision: 1. total *n* = 50–100 2. total *n* < 50
Very Low (We have very little confidence in the measurement property estimate: the true measurement property is likely to be substantially different from the estimate of the measurement property	Indirectness: 1. Serious 2. Very serious

Starting point: assumption that the evidence is of high quality. Information on how to downgrade is described in the COSMIN user manual [31]. Definitions were adapted from the GRADE approach [11]. *n* = sample size

#### Generating recommendations for the use of PROMs in patients with hyperhidrosis

Each assessed instrument was assigned to a recommendation category according to its methodological quality and adequacy. We used three categories of recommendation that were proposed by the COSMIN group [36]:

A. PROMs with evidence for sufficient content validity (any level) and at least low-quality evidence for sufficient internal consistency.
B. PROMs categorized not in A or C.
C. PROMs with high-quality evidence for an insufficient measurement property

PROMs of category A can be recommended for use and results obtained with these PROMs can be seen as trustworthy. For PROMs of category B, further validation is needed; however, they still have the opportunity to be recommended for use. PROMs of category C should not be recommended for use. If only PROMs of category B are found, the PROM with the best evidence for content validity can be preliminarily recommended for use, until further evidence is given [31].

Our aim was to identify the best (currently available) PROM(s) in hyperhidrosis.

## Results

**Fig. 1:**
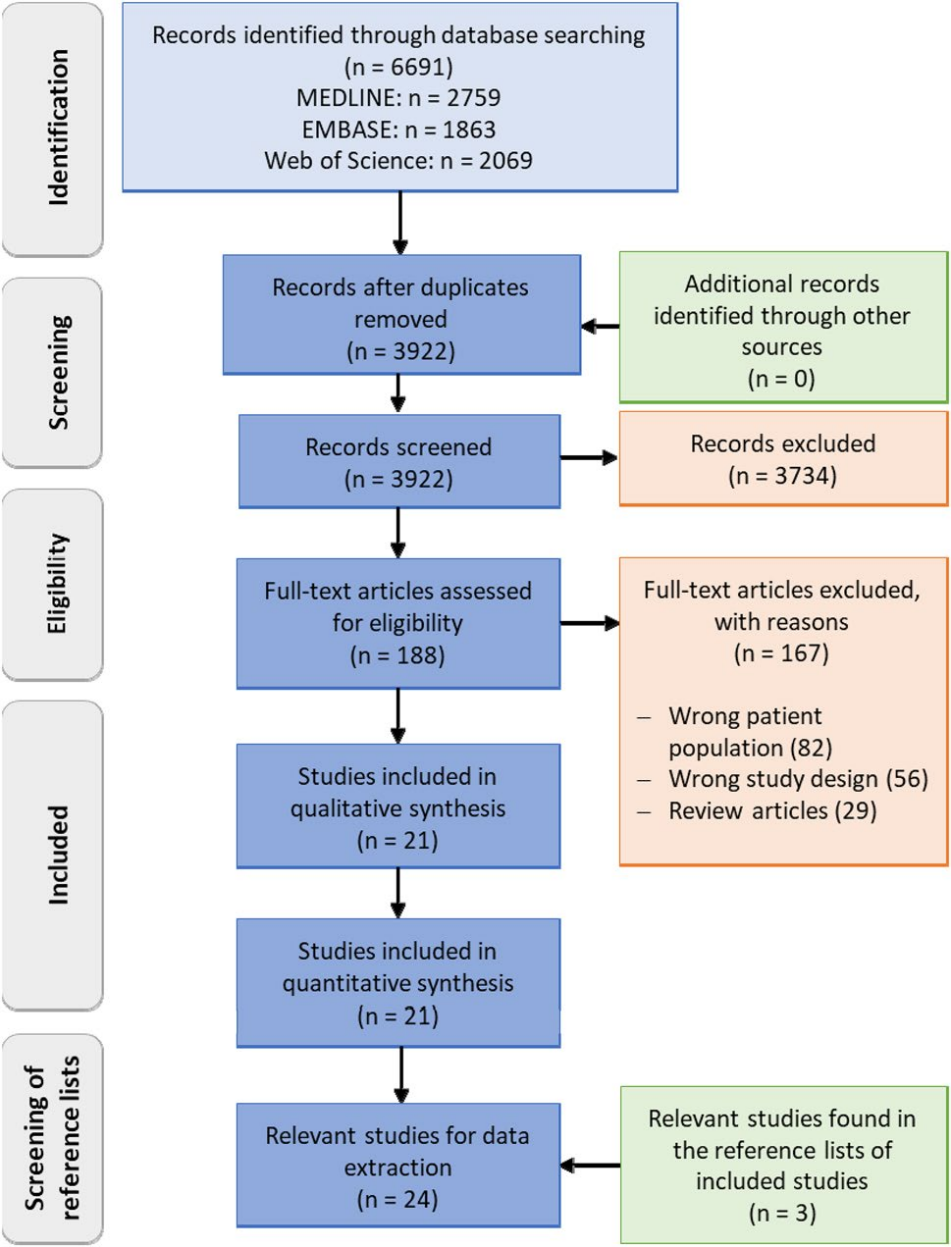
Adapted PRISMA 2009 flow diagram [29]. For more information, visit www.prisma-statement.org. *PROM* patient-reported outcome measure

Searching the bibliographic databases yielded 6691 records of which 3922 remained after deduplication and were moved into the screening. We found 188 studies to be included in the full-text screening, 19 of which were considered for qualitative and quantitative synthesis (Fig. 1). Three further relevant articles were found in the reference lists of the included studies, resulting in 24 relevant studies for data extraction. One study by Kamudoni contained data on the content validity of the HidroQoL, but did not formally meet the inclusion criteria [19]. Nevertheless, supplementary information on content validity was extracted to assess the methodological quality of the PROM development. The PhD thesis of Paul Kamudoni was also included as it provided complementary information to the paper by Kamudoni et al*.* published in 2015 [18, 20]. The development study of Amir et al*.* [1] was added since it provided preliminary work for the development of the Hyperhidrosis Quality of Life Questionnaire (HQLQ) by de Campos et al*.* [6]. In total, we identified 24 studies reporting on 13 different PROMs.

Five included studies reported on the DLQI [3, 26, 41, 43, 46]; three on the HQLQ [1, 6, 35], the HidroQoL [9, 18, 20] and the HDSS [39, 46, 47], respectively; two on the SF-36 [38, 46], the Hyperhidrosis Disease Severity Measure-Axillary (HDSM-Ax) [16, 22], as well as the Hyperhidrosis Scale (HS) [21, 35]; and one each on the ASDD and its child version (ASDD-C) [34], the Hyperhidrosis Impact Questionnaire (HHIQ) [25], the Illness Intrusiveness Rating Scale (IIRS) [5], the Hyperhidrosis Questionnaire (HQ) [24], the Sweating Cognitions Inventory (SCI) [52] and the self-evaluation scale (SES) [23]. The questionnaires of Amir et al*.* [1] and de Campos et al*.* [6] were also found as two independent questionnaires. Since the de Campos questionnaire is a further development based on the main content of the study of Amir et al*.* [1], studies on these two questionnaires were summarized in this paper under the de Campos questionnaire. Additionally, we found several designations for the questionnaires of de Campos et al*.* [6] and Kuo et al*.* [24]. In the following, we adopted the terms used by Wade et al*.* [50], namely the HQLQ and HQ.

### Data extraction

Regarding the data extraction using the COSMIN Risk of Bias checklist, the two reviewers had an agreement of 80.35%. Consensus was mostly found between the two reviewers. Some major disagreements were discussed with a third reviewer having expertise with the COSMIN methodology.

#### Evaluation of content validity

An ‘inadequate’ PROM development rating was found for three PROMs: the HQ, the HQLQ as well as the SCI. Regarding the HQLQ, the omission of a cognitive interview study or pilot test to assess the comprehensibility and comprehensiveness in a sample representing the target population was the reason for the ‘inadequate’ rating. The evaluation of the HQ’s and the SCI’s PROM development was inadequate since the PROM development studies were based on a literature search only or performed in a group of clinicians and researchers rather than in a sample representing the target population for which the PROM was developed, as required. All content validity studies, when conducted, were of doubtful quality as information on the number of researchers involved in the data analysis was mostly lacking (Table 4).

**Table 4:** COSMIN Risk of Bias overall ratings for content validity

	ASDD/ASDD-C	HDSM-Ax	HidroQoL	HQ	HQLQ	SCI
Box 1. PROM development	Doubtful	Doubtful	Doubtful	Inadequate	Inadequate	Inadequate
Box 2. Content validity	–	Doubtful	Doubtful	Doubtful	–	–

*COSMIN* COnsensus-based Standards for the selection of health Measurement Instrument, *PROM* patient-reported outcome measure, *ASDD(-C)* Axillary Sweating Daily Diary (Child version), *HDSM-Ax* Hyperhidrosis Disease Severity Measure-Axillary, *HidroQoL* Hyperhidrosis Quality of Life Index, *HQ* Hyperhidrosis Questionnaire, *HQLQ* Hyperhidrosis Quality of Life Questionnaire, *SCI* Sweating Cognitions Inventory

The quality of evidence of the HidroQoL was moderate since at least one content validity study of doubtful quality was available [18, 20]. Copies of the HQ and SES were not available. Only the general design and the structure of the response options were known, but no complete version of the questionnaires and their wording was available. Therefore, some aspects were judged as “?” in the reviewer’s rating. In case of an inconsistent or indeterminate overall rating, there was no grading of the quality of the evidence (Table 5).

**Table 5:** Content validity rating of the included PROMs

		Relevance	Comprehensiveness	Comprehensibility	Content validity rating
ASDD/ASDD-C	Overall rating	+	−	+	Inconsistent ( ±)
	Quality of evidence	No grading if overall rating is inconsistent			
DLQI	Overall rating	±	–	±	Insufficient ( −)
	Quality of evidence	Very low (due to risk of bias)			
HDSM-Ax	Overall rating	±	+	+	Sufficient ( +)
	Quality of evidence	Low (due to risk of bias and inconsistency)			
HDSS	Overall rating	±	–	–	Insufficient ( −)
	Quality of evidence	Very low (due to risk of bias and inconsistency)			
HHIQ	Overall rating	+	+	±	Sufficient ( +)
	Quality of evidence	Very low (due to risk of bias and inconsistency)			
HidroQoL	Overall rating	+	+	+	Sufficient ( +)
	Quality of evidence	Moderate (due to risk of bias)			
HQ	Overall rating	±	+	+	Sufficient ( +)
	Quality of evidence	Very low (due to risk of bias and inconsistency)			
HQLQ	Overall rating	+	+	+	Sufficient ( +)
	Quality of evidence	Very low (due to risk of bias)			
HS	Overall rating	±	–	+	Inconsistent ( ±)
	Quality of evidence	No grading if overall rating is inconsistent			
IIRS	Overall rating	±	±	±	Inconsistent ( ±)
	Quality of evidence	No grading if overall rating is indeterminate			
SCI	Overall rating	+	+	+	Sufficient ( +)
	Quality of evidence	Very low (due to risk of bias)			
SES	Overall rating	?	+	?	Indeterminate (?)
	Quality of evidence	No grading if overall rating is indeterminate			
SF-36	Overall rating	±	−	+	Inconsistent ( ±)
	Quality of evidence	No grading if overall rating is inconsistent			

*PROMs* patient-reported outcome measures, *ASDD(-C)* Axillary Sweating Daily Diary (Child version), *DLQI* Dermatology Life Quality Index, *HDSM-Ax* Hyperhidrosis Disease Severity Measure-Axillary, *HDSS* Hyperhidrosis Disease Severity Scale, *HHIQ* Hyperhidrosis Impact Questionnaire, *HidroQoL* Hyperhidrosis Quality of Life Index, *HQ* Hyperhidrosis Questionnaire, *HQLQ* Hyperhidrosis Quality of Life Questionnaire, *HS* Hyperhidrosis Scale, *IIRS* Illness Intrusiveness Rating Scale, *SCI* Sweating Cognitions Inventory, *SES* self-evaluation scale, *SF-36* short-form health survey (with 36 items)

We could not find high-quality evidence that the content validity of any PROM was insufficient; thus, the remaining measurement properties of every PROM were further assessed.

#### Evaluation of the remaining measurement properties (structural validity, internal consistency, cross-cultural validity/measurement invariance, reliability, measurement error, criterion validity, hypotheses testing for construct validity and responsiveness)

In total, the methodological quality of 111 measurement properties was rated. 44 measurement properties (39.6%) had very good, 25 (22.5%) had adequate and 21 (18.9%), respectively, doubtful and inadequate methodological quality (Table 6).

**Table 6:** COSMIN Risk of Bias overall ratings for the remaining measurement properties

PROMs	ASDD/ *ASDD-C* ^a^	DLQI ^b,c,d,e,f^	HDSM-Ax^g,h^	HDSS^d,i,j^	HHIQ^k^	HidroQoL^l,m,n,o^	HQ^p^	HQLQ^q,r,s^	HS^r,t^	IIRS^u^	SCI^v^	SES^w^	SF-36^d,n^
Structural validity			Inadequate			Very good	Inadequate				Adequate		
			Inadequate			Adequate					Very good		
			Very good			Very good							
Internal consistency			Very good			Very good	Very good	Doubtful		Very good	Very good		
						Very good					Very good		
						Very good							
Reliability	Doubtful		Doubtful	Doubtful		Adequate				Doubtful			
	*Doubtful*			Doubtful		Adequate							
	Doubtful					Adequate							
Criterion validity									Very good		Very good	Very good	
									Very good				
Hypotheses testing for construct validity	Very good	Very good	Adequate	Doubtful		Very good		Adequate	Adequate	Inadequate	Very good	Adequate	Doubtful
	*Adequate*	Doubtful	Very good	Doubtful		Very good		Very good	Inadequate	Doubtful	Adequate	Adequate	Very good
	Very good	Inadequate	Inadequate	Doubtful		Adequate			Adequate		very good		
	Adequate	Adequate	Adequate	Inadequate		Adequate			Inadequate				
	Inadequate					Inadequate			Adequate				
	Inadequate					Inadequate							
	*Inadequate*					Very good							
	Inadequate					Inadequate							
	Doubtful					Adequate							
	*Doubtful*												
	Doubtful												
Responsiveness	Very good	Very good	Very good	Very good	Very good	Very good		Very good	Very good	Doubtful			Doubtful
	*Adequate*	Very good		Adequate		Very good		Doubtful					Very good
	Very good	Very good		Adequate		Adequate							
	Adequate	Very good				Very good							
	Inadequate	Very good				Very good							
	Inadequate					Inadequate							
	*Inadequate*												
	Inadequate												
	Doubtful												

*COSMIN* COnsensus-based Standards for the selection of health Measurement Instrument, *PROMs* patient-reported outcome measures, *ASDD(-C)* Axillary Sweating Daily Diary (Child version), *DLQI* Dermatology Life Quality Index, *HDSM-Ax* Hyperhidrosis Disease Severity Measure-Axillary, *HDSS* Hyperhidrosis Disease Severity Scale, *HHIQ* Hyperhidrosis Impact Questionnaire, *HidroQoL* Hyperhidrosis Quality of Life Index, *HQ* Hyperhidrosis Questionnaire, *HQLQ* Hyperhidrosis Quality of Life Questionnaire, *HS* Hyperhidrosis Scale, *IIRS* Illness Intrusiveness Rating Scale, *SCI* Sweating Cognitions Inventory, *SES* self-evaluation scale, *SF-36* short-form health survey (with 36 items)

^a^Nelson (2019)

^b^Swartling (2001)

^c^Campanati (2003)

^d^Tetteh (2009)

^e^Skroza (2011)

^f^Lynch (2020)

^g^Kirsch (2018)

^h^Hobart (2021)

^i^Varella (2016)

^j^Sener (2019)

^k^Li (2018)

^l^Kamudoni (2014)

^m^Kamudoni (2015)

^n^Schreiner (2019)

^o^Gabes (2020)

^p^Kuo (2004)

^q^Amir (2000)

^r^Panhofer (2006)

^s^de Campos (2016)

^t^Keller (2009)

^u^Cina (1999)

^v^Wheaton (2011)

^w^Krogstad (2005)

### Characteristics of the included PROMs and study populations

A complete overview of all included PROMs is presented in Appendix 3 (supplementary files). Characteristics of the included study populations are shown in Appendix 4 (supplementary files).

Sample sizes ranged from 8 to 665 patients. Only four [1, 34, 43, 46] of the 21 studies included children < 16 years. On average, slightly more women participated in the studies, accounting for 64% of the study populations. The studies were classified into development, validation and intervention studies and were conducted in more than 15 countries. The lowest number of items in a questionnaire was one, the highest 41 with an optional 10-item follow-up module. Only two questionnaires partially use a dichotomous response format, whilst the predominant Likert scale format is applied in various forms (3- to 11-point Likert scale) in all PROMs.

Most of the PROMs are disease-specific measurement instruments for hyperhidrosis. The ASDD(-C) and the HDSM-Ax are also site-specific for axillary hyperhidrosis. The same applies to the HS and the SES for palmar hyperhidrosis. These four PROMs, as well as the HDSS for primary hyperhidrosis in general, intend to measure the sweating severity. The HidroQoL, HQ and HQLQ, on the other hand, are disease-specific PROMs measuring health-related QoL. Similarly, the HHIQ measures the impact of hyperhidrosis in the different domains in life. However, the SCI takes a different approach and captures the types of dysfunctional negative beliefs in hyperhidrosis held by patients.

The remaining PROMs, the DLQI, the IIRS and the SF-36, are not hyperhidrosis-specific. The SF-36 is a measurement instrument of generic health-related QoL. The DLQI is applied to patients with skin conditions and measures the impact of the condition on the patient's QoL. A child version is also available. The IIRS is validated for patients with moderate to severe chronic disabling and/or life-threatening diseases. The construct of intrusiveness represents the disruptive effects on various aspects of life due to the disease. As general questionnaires, these PROMs are applicable in a large population, which is also shown by the high number of translated versions. The DLQI is available in more than 110 languages (https://www.cardiff.ac.uk/medicine/resources/quality-of-life-questionnaires/dermatology-life-quality-index). There are various validated translations for the SF-36 (https://www.rand.org/health-care/surveys_tools/mos/36-item-short-form.html), such as German, French or Japanese and also the IIRS has been translated into various languages, e.g. French or Chinese [7].

### Information on interpretability and feasibility

Information on the distribution of scores in the study population was only given for the HidroQoL and the SCI. The results in the thesis of Kamudoni [18] showed a positive skew for the items towards higher response categories, whereas Gabes et al*.* [9] indicated negative skewness and evidence that the data were not normally distributed. Both reports showed ceiling effects for most of the items. According to Gabes et al*.* [9], 26–91% of the patients chose the highest response category. Floor effects were found by Kamudoni [18] for 13 items of the HidroQoL. Wheaton et al*.* [52] compared the distribution of scores in patients with hyperhidrosis and in a control group, showing a normal distribution for patients and positively skewed scores for controls. Small floor and ceiling effects were observed for the HDSM-Ax [16]. Other ceiling effects were only given by Nelson et al*.* [34] indicating some ceiling effects for Item 4 of the ASDD. The distribution of missing items was analysed for the HidroQoL, showing an increase in missing data towards the end of the questionnaire. However, no further structure in the missing data was apparent. Minimal important difference (MID) values of 3 to 4 were proposed for the HidroQoL by Kamudoni [18] and Gabes et al*.* [9]; differences in values are likely due to a more homogenous study population in the latter study. Hobart et al*.* [16] estimated meaningful change scores of the HDSM-Ax and stated that a change in the HDSM-Ax total score of one point represents a clinically meaningful change in axillary hyperhidrosis severity. Nelson et al*.* [34] did not calculate a MID value but referred to patients who achieved a reduction in weekly average scores on ASDD Item 2 of ≥ 4 points as responders to treatment. No information on response shift could be extracted of the included studies.

All PROMs are self-administered. There were no problems reported regarding the patient's comprehensibility or administration. Campanati et al*.* [3] explicitly mentioned that the DLQI does not require any specific intellectual abilities. For most questionnaires, it was stated that they have a short completion time, ranging from only a few minutes for the DLQI to approximately 8–10 min for the HQ. The HDSS can also be used by non-specialists. The HDSS and the SES are only single-item instruments. For the DLQI, HDSM-Ax, HidroQoL, HQLQ, IIRS and SCI, a simple summary score can be obtained by adding up all item scores. For the ASDD(-C), consisting of 2 to 4 questions, scores for the individual items can be calculated. For the HQ and SF-36, scores for their individual domains can be obtained. Only for the HS, a normalized score can be used by dividing the total score by the number of completed items. No information on the scoring system was given for the HHIQ. The DLQI is copyrighted, but can be used without further permission for routine clinical purposes (https://www.cardiff.ac.uk/medicine/resources/quality-of-life-questionnaires/dermatology-life-quality-index). Two other questionnaires are copyrighted, the ASDD(-C) and the HidroQoL, but no information about their terms of use was found. The SF-36 is freely available on the RAND homepage and, except for a credit line, requires no further permission for use (https://www.rand.org/health-care/surveys_tools/mos/36-item-short-form.html). A copy of the HHIQ was kindly provided by the responsible company for an evaluation within this paper. For the remaining PROMs, no information could be retrieved regarding their accessibility.

#### Summary of findings (SoF) tables and recommendation

The summarized results per measurement property per PROM are presented in Table 7. The overall ratings for reliability of the HDSS and for hypotheses testing for construct validity of the ASDD-C and the SCI were inconsistent since not all studies reported ICCs ≥ 0.7 for the HDSS and only around half of the a priori hypotheses could be confirmed for the ASDD-C and the SCI. Structural validity and internal consistency of the SCI were downgraded due to indirectness since one relevant study was partly performed in another population of interest (student population). The HQLQ and the SF-36 showed inconsistent results regarding hypotheses testing for construct validity. We decided to base the overall rating on the majority of the results and therefore downgraded the quality of evidence for one level due to inconsistency. For the items 3 and 4 of the ASDD, we found an inconsistent overall rating since only one-third of the a priori hypotheses could be confirmed with the data extracted. There was one study in which test–retest reliability was assessed twice for the HidroQoL, with and without an intervention between the two measurements. Results were presented for both subgroups separately. The insufficient reliability rating found for the intervention-subgroup might be explained by treatment effects and should not be overestimated. Furthermore, for the HidroQoL and the HDSM-Ax, very few hypotheses for construct validity and responsiveness could not be confirmed. However, the corresponding correlations were only 0.03–0.09 above the fixed threshold and therefore not classified as “inconsistent”.

**Table 7:** Summary of Findings (SoF) Tables

PROMs	Summary or pooled result	Overall rating	Quality of evidence
*Structural validity*			
HDSM-Ax	No monotonicity, local dependence, degrees of model misfit	Insufficient	High
HidroQoL	Draft 21-item HidroQoL: CFI = 0.98, TLI = 0.977, RMSEA = 0.077 Final 18-item HidroQoL: CFI = 0.815, RMSEA = 0.084, SRMR = 0.074	Sufficient	High
HQ	Model fit not reported	Indeterminate	–
SCI	CFI = 0.97, TLI = 0.96, SRMR = 0.03, RMSEA = 0.077	Sufficient	Moderate (due to indirectness)
*Internal consistency*			
HDSM-Ax	Criteria for “at least low evidence for sufficient structural validity” not met	Indeterminate	–
HidroQoL	Total scale: 0.89–0.90, domain 1: 0.76–0.81, domain 2: 0.86–0.87, *n* = 764	Sufficient	High
HQ	0.71–0.95, *n* = 85	Sufficient	Moderate (due to imprecision)
HQLQ	0.84, *n* = 34–48	Sufficient	Very low (due to risk of bias and imprecision)
IIRS	0.80, n = 80	Sufficient	Moderate (due to imprecision)
SCI	0.91–0.92, *n* = 708	Sufficient	Moderate (due to indirectness)
*Reliability*			
ASDD	Item 2: 0.91–0.94, item 3: 0.89–0.90, item 4: 0.88–0.89, *n* = 770	Sufficient	Moderate (due to risk of bias)
ASDD-C	Item 2: 0.92, *n* = 32	Sufficient	Very low (due to risk of bias and imprecision)
HDSM-Ax	0.543, *n* = 227	Insufficient	Low (due to risk of bias)
HDSS	0.65–0.84, *n* = 92	Inconsistent	–
HidroQoL	*Without intervention (subgroup)* total scale: 0.93, domain 1: 0.88–0.89, domain 2: 0.91–0.92	Sufficient	High
	*with intervention (subgroup)* total scale: 0.61, domain 1: 0.53, domain 2: 0.66	Insufficient	Moderate (due to risk of bias)
IIRS	0.89, n = 68	Sufficient	Very low (due to imprecision)
*Criterion validity*			
HS (#1, #2)	AUC not reported	Indeterminate	–
SCI	0.80, *n* = 708	Sufficient	High
SES	0.93, *n* = 34	Sufficient	Low (due to imprecision)
*Hypotheses testing for construct validity*			
ASDD	Item 2: 7 out of 8 hypotheses confirmed, *n* = 770 Item 3: 6 out of 8 hypotheses confirmed, *n* = 802 Item 4: 6 out of 8 hypotheses confirmed, *n* = 802	Sufficient (inadequate studies ignored)	High
ASDD-C	Item 2: 2 out of 4 hypotheses confirmed, *n* = 32	Inconsistent (inadequate studies ignored)	–
DLQI	3 out of 4 hypotheses confirmed, *n* = 171	Sufficient (inadequate study ignored)	High
HDSM-Ax	4 out of 5 hypotheses confirmed, n = 261	Sufficient (inconsistency could be explained)	High
HDSS	1 out of 1 hypothesis confirmed, *n* = 369	Sufficient (inadequate study ignored)	Moderate (due to risk of bias)
HidroQoL	12 out of 14 hypotheses confirmed, *n* = 329–333	Sufficient (inconsistency could be explained)	High
HQLQ	1 out of 3 hypotheses confirmed, *n* = 144–160	Insufficient (based on majority of the results)	Moderate (due to inconsistency)
HS	1 out of 1 hypothesis confirmed, *n* = 132–146	Sufficient (inadequate study ignored)	high
IIRS	1 out of 1 hypothesis confirmed, *n* = 80	Sufficient (inadequate study ignored)	Very low (due to risk of bias and imprecision)
SCI	3 out of 8 hypotheses confirmed, *n* = 708	Inconsistent	–
SES	2 out of 2 hypotheses confirmed, *n* = 34	Sufficient	Low (due to imprecision)
SF-36	1 out of 3 hypotheses confirmed, *n* = 184	Insufficient (based on the high-quality study)	Moderate (due to inconsistency)
*Responsiveness*			
ASDD	Item 2: 3 out of 4 hypotheses confirmed, n = 802	Sufficient (inadequate studies ignored)	High
	Item 3: 1 out of 3 hypotheses confirmed, *n* = 802 Item 4: 1 out of 3 hypotheses confirmed, *n* = 802	Inconsistent (inadequate studies ignored)	–
ASDD-C	Item 2: 3 out of 3 hypotheses confirmed, *n* = 32	Sufficient (inadequate studies ignored)	Very low (due to risk of bias and imprecision)
DLQI	5 out of 5 hypotheses confirmed, *n* = 167	Sufficient	High
HDSM-Ax	1 out of 1 hypothesis confirmed, *n* = 201	Sufficient	High
HDSS	3 out of 3 hypotheses confirmed, *n* = 307	Sufficient	High
HHIQ	1 out of 1 hypothesis confirmed, *n* = 106	Sufficient	High
HidroQoL	6 out of 7 hypotheses confirmed, *n* = 433–444	Sufficient (inconsistency could be explained)	High
HQLQ	1 out of 1 hypothesis confirmed, *n* = 509	Sufficient	High
HS	1 out of 1 hypothesis confirmed, *n* = 106	Sufficient	High
IIRS	1 out of 1 hypothesis confirmed, *n* = 4	Sufficient	Very low (due to risk of bias and imprecision)
SF-36	0 out of 2 hypotheses confirmed, *n* = 120	Insufficient	High

*PROMs* patient-reported outcome measures, *ASDD(-C)* Axillary Sweating Daily Diary (Child version), *DLQI* Dermatology Life Quality Index, *HDSM-Ax* Hyperhidrosis Disease Severity Measure-Axillary, *HDSS* Hyperhidrosis Disease Severity Scale, *HHIQ* Hyperhidrosis Impact Questionnaire, *HidroQoL* Hyperhidrosis Quality of Life Index, *HQ* Hyperhidrosis Questionnaire, *HQLQ* Hyperhidrosis Quality of Life Questionnaire, *HS* Hyperhidrosis Scale, *IIRS* Illness Intrusiveness Rating Scale, *SCI* Sweating Cognitions Inventory, *SES* self-evaluation scale, *SF-36* short-form health survey (with 36 items), *CFI* Comparative Fit Index, *TLI* Tucker-Lewis Index, *RMSEA* Root Mean Square Error of Approximation, *SRMR* Standardized Root Mean Square Residual, *n* sample size, *AUC* area under the curve

The results of the SoF Tables were used to recommend the most appropriate PROM. The final recommendations according to the COSMIN guidelines [31] for all four PROMs are presented in Table 8.

**Table 8:** Recommendations for use in future hyperhidrosis trials

PROM	Category A		Category C	
	Sufficient content validity (any level)	At least low-quality evidence for sufficient internal consistency	High quality evidence for an insufficient measurement property	Recommen-dation
ASDD/ASDD-C	✗	✗	✗	B
DLQI	✗	✗	✗	B
HDSM-Ax	✓	✗	✓	C
HDSS	✗	✗	✗	B
HHIQ	✓	✗	✗	B
HidroQoL	✓	✓	✗	A
HQ	✓	✓	✗	A
HQLQ	✓	✗	✗	B
HS	✗	✗	✗	B
IIRS	✗	✓	✗	B
SCI	✓	✓	✗	A
SES	✗	✗	✗	B
SF-36	✗	✗	✓	C

*PROM* patient-reported outcome measures, *ASDD(-C)* Axillary Sweating Daily Diary (Child version), *DLQI* Dermatology Life Quality Index, *HDSM-Ax* Hyperhidrosis Disease Severity Measure-Axillary, *HDSS* Hyperhidrosis Disease Severity Scale, *HHIQ* Hyperhidrosis Impact Questionnaire, *HidroQoL* Hyperhidrosis Quality of Life Index, *HQ* Hyperhidrosis Questionnaire, *HQLQ* Hyperhidrosis Quality of Life Questionnaire, *HS* Hyperhidrosis Scale, *IIRS* Illness Intrusiveness Rating Scale, *SCI* Sweating Cognitions Inventory, *SES* self-evaluation scale, *SF-36* short-form health survey (with 36 items)

## Discussion

This systematic review provides a first synthesis of the methodological assessment of the measurement properties of 13 PROMs used in patients with hyperhidrosis following an established methodology. As Wade et al*.* [50] have already stated in their review in 2017, a high level of information about the development and the validation of PROMs is necessary to be able to appropriately judge them. In this study, three PROMs, the HidroQoL, the HQ and the SCI, showed evidence for content validity and moderate- to high-quality of evidence for internal consistency and therefore can be further recommended for use according to the COSMIN criteria. Results obtained with these PROMs can be seen as trustworthy. Those PROMs are assessing different constructs. The HidroQoL and the HQ are both measuring health-related QoL, whereas the SCI is measuring sweating cognitions, i.e. types of dysfunctional negative beliefs in hyperhidrosis.

Especially construct validity of the SCI should be further assessed since we found an inconsistent overall rating for this measurement property. An evaluation of the responsiveness and reliability of the SCI is also needed, as there are still gaps in evidence. In addition, further validation studies should be conducted within the target population (patients with hyperhidrosis) to strengthen the quality of evidence and avoid downgrading due to indirectness. Regarding the two QoL-PROMs, the HidroQoL currently seems to be more convincing than the HQ. This is based on a higher quality of evidence of the HidroQoL regarding content validity and internal consistency as well as a larger study population where these results are based on. Moreover, the HidroQoL lacked only evaluations of three measurement properties, measurement error, criterion validity and cross-cultural validity. Many other measurement properties can already be considered as sufficient on a high quality of evidence level. The HQ met the requirements for a recommendation according to the COSMIN criteria; however, evidence gaps remain, for instance with regard to structural validity with an indeterminate overall rating. Especially in clinical trials, PROMs should be reliable, valid, responsive and feasible and therefore, a comprehensive assessment of the measurement properties is crucial.

Many other PROMs such as the ASDD(-C), DLQI, HDSS, HHIQ, HQLQ, HS, IIRS and the SES could still possibly be recommended for use, but further validations studies are needed. The HDSM-Ax and the SF-36 cannot be recommended for use in patients with hyperhidrosis since we found moderate- and high-quality evidence for insufficient measurement properties (structural validity for the HDSM-Ax and construct validity and responsiveness for the SF-36). The HDSM-Ax did not fit the Rasch model what was shown in two independent studies and what led to an insufficient structural validity rating. The poor performance of the SF-36 could also be a consequence of the fact that generic as well as skin-specific PROMs do not comprehensively reflect the specific needs of patients with hyperhidrosis. This assumption is also reflected for instance in the insufficient content validity rating of the DLQI. Importantly, future validation studies should look at the interpretability and feasibility of PROMs since only little information was available for the currently included PROMs.

### Strengths and limitations of this systematic review

In this systematic review, we identified several strengths: an a priori registered protocol, the use of a comprehensive and sensitive search filter, the search in three large databases (MEDLINE, EMBASE and Web of Science), several smaller databases and reference lists of the included studies, the application of predefined eligibility criteria and the use of the COSMIN Risk of Bias checklist to assess the methodological quality of the included studies. Two independent reviewers (MG und GK) carried out every step of the review process to ensure consistency. One eligible paper where two of the authors (MG and CA) were conflicted was evaluated by two unconflicted reviewers (GK and CT). Discrepancies were discussed and resolved within the whole research team. A potential limitation of this systematic review is the fact that not all reference lists of relevant full-texts were searched for further eligible studies (backward search). We have not performed a forward search either.

## Conclusion

This systematic review suggests that currently three PROMs, the HidroQoL, the HQ and the SCI, can be recommended for use in patients with hyperhidrosis. To strengthen and extend the evidence of those measurement instruments, future validation studies should focus on those PROMs.

## Data Availability

All supplementary files and additional data that support the findings of this study are available from the University of Regensburg Publication Server (10.5283/epub.46453, https://epub.uni-regensburg.de/46453/).

## Code availability

All data analyses were conducted using the COSMIN manuals (available at https://www.cosmin.nl/tools/guideline-conducting-systematic-review-outcome-measures/).
